# OpenFLUX: efficient modelling software for ^13^C-based metabolic flux analysis

**DOI:** 10.1186/1475-2859-8-25

**Published:** 2009-05-01

**Authors:** Lake-Ee Quek, Christoph Wittmann, Lars K Nielsen, Jens O Krömer

**Affiliations:** 1 Australian Institute for Bioengineering and Nanotechnology, The University of Queensland, QLD 4072, Australia; 2 Institute of Biochemical Engineering, Technische Universität Braunschweig, 38106 Braunschweig, Germany

## Abstract

**Background:**

The quantitative analysis of metabolic fluxes, i.e., *in vivo *activities of intracellular enzymes and pathways, provides key information on biological systems in systems biology and metabolic engineering. It is based on a comprehensive approach combining (i) tracer cultivation on ^13^C substrates, (ii) ^13^C labelling analysis by mass spectrometry and (iii) mathematical modelling for experimental design, data processing, flux calculation and statistics. Whereas the cultivation and the analytical part is fairly advanced, a lack of appropriate modelling software solutions for all modelling aspects in flux studies is limiting the application of metabolic flux analysis.

**Results:**

We have developed OpenFLUX as a user friendly, yet flexible software application for small and large scale ^13^C metabolic flux analysis. The application is based on the new Elementary Metabolite Unit (EMU) framework, significantly enhancing computation speed for flux calculation. From simple notation of metabolic reaction networks defined in a spreadsheet, the OpenFLUX parser automatically generates MATLAB-readable metabolite and isotopomer balances, thus strongly facilitating model creation. The model can be used to perform experimental design, parameter estimation and sensitivity analysis either using the built-in *gradient-based search *or *Monte Carlo *algorithms or in user-defined algorithms. Exemplified for a microbial flux study with 71 reactions, 8 free flux parameters and mass isotopomer distribution of 10 metabolites, OpenFLUX allowed to automatically compile the EMU-based model from an Excel file containing metabolic reactions and carbon transfer mechanisms, showing it's user-friendliness. It reliably reproduced the published data and optimum flux distributions for the network under study were found quickly (<20 sec).

**Conclusion:**

We have developed a fast, accurate application to perform steady-state ^13^C metabolic flux analysis. OpenFLUX will strongly facilitate and enhance the design, calculation and interpretation of metabolic flux studies. By providing the software *open source*, we hope it will evolve with the rapidly growing field of fluxomics.

## Background

Metabolic flux analysis (MFA) plays a central role in metabolic engineering and systems biology [1]. Metabolic fluxes most closely reflect the underlying metabolic phenotype, whereas other 'omics approaches only yield a sense of metabolic capacities (transcriptomics/proteomics) or thermodynamic driving forces (metabolomics). Metabolic flux analysis is particular important in rational strain engineering, where we specifically seek to manipulate the metabolic phenotype.

Due to the high complexity of the examined metabolic network, flux analysis typically involves the use of a stoichiometric model, in which the metabolic reactions available to the cell are parameterized before the fluxes are estimated from experimental data [2]. State-of-art flux analysis today includes the use of stable isotopes to overcome problems such as incomplete resolution of important cellular pathways or the need to rely on stoichiometric parameters with high uncertainty such as ATP yield (Y_x/ATP_) or P/O ratio which are inherently linked to the purely stoichiometric approaches [3]. ^13^C-based MFA therefore is a powerful extension of MFA [3]. In such studies, after feeding ^13^C-labelled substrate(s), one measures the ^13^C tracer enrichment patterns of metabolites that are rich in flux information, using instruments such as nuclear magnetic resonance spectroscopy (NMR) [4,5] or mass spectrometry (MS) [6]. There are mainly two different approaches to extract flux information from the labelling patterns: by model-based flux fitting [3], and by analytical interpretation of flux ratios [7] (both approaches briefly reviewed in [8]). Redundant pathways that contribute differently to tracer distribution can thus be resolved. Flux analysis is carried out independently from energy and redox balancing, because the balancing equations only involve the carbon backbone. Conversely, the flux results can be used to check the consistency of energy and redox balances [9].

There has been significant development especially concerning the experimental framework for ^13^C MFA [10]. ^13^C MFA has been applied to various prokaryotic and eukaryotic systems [11-13] involving miniaturized screening studies in small scale [14,15]. There is an increasing trend towards large-scale network-based stationary ^13^C MFA [16,17], as well as non-stationary (i.e., dynamic) ^13^C MFA [18-20]. Large-scale metabolic models are preferred in order to capture, as many reactions as possible, bearing effects on carbon labelling, and to maintain global consistency of flux estimates. Considering metabolism in isolated parts or using overly summarized metabolic models can lead to biased results [17]. However, specifying large sets of isotopomer balances and subsequently performing parameter estimation can be very cumbersome.

Several software packages have been developed to facilitate flux analysis, the most popular being FiatFlux [21] and 13C-FLUX [22]. FiatFlux implements the flux ratio approach to ^13^C MFA [7] and comes preconfigured to derive flux ratios and net fluxes for [1-^13^C]- and [U-^13^C]-glucose experiments and GC-MS analysis of proteinogenic amino acids for several microorganisms. Recent developments allow to generate equation systems automatically [23], which facilitates the extension of the flux ratio approach to various metabolic models, input substrates and labelling data.

In contrast, 13C-FLUX is a general purpose package for modelling, simulation, design, evaluation, and statistical analysis of ^13^C-labelling experiments [22]. Unfortunately, 13C-FLUX is relatively cumbersome to use in terms of requiring the user to specify free fluxes, to set up the initial solution, and to manually initialize and terminate each optimization. It is not possible to perform multiple rounds of optimization unsupervised, which is frequently used to check convergence of the optimization results. There is a general lack of support in aspects of experimental design, i.e., explore change in labelling patterns for a different flux distribution and/or various combinations of input substrates. For expert users, there is limited opportunity to modify source code for implementation of new algorithms and workflows for different labelling problems.

There is a need for ^13^C MFA tool that is simple, flexible and transparent. Fast computation is crucial. For a non-expert user, the software must enable a smooth reproducible workflow covering the whole process from metabolic model definition to flux estimation. A flexible approach necessarily supports user-defined metabolic systems, while a transparent computational model offers expert users the opportunity to tailor make downstream algorithms for parameter estimation and statistical analysis.

To meet this challenge, we have developed OpenFLUX, a simple yet flexible application to perform steady-state ^13^C MFA using mass isotopomer distribution data. OpenFLUX provides the user a versatile and intuitive spreadsheet-based interface to control the underlying metabolite and isotopomer balance models used for flux analysis and allowing for the implementation of large-scale metabolic networks. The user then has the option of using the accompanying algorithm package for flux estimation and sensitivity analysis, or applying alternative numerical approaches for flux analysis (e.g., [24]). OpenFLUX generates isotopomer balance model based on the EMU decomposition algorithm [25]. Using EMU variables is computationally more efficient because the number of necessary isotopomer balances is significantly reduced [25] compared to alternative representations of labelling distribution of metabolites, such as AAV (atom activity vector) [26], IDV (isotopomer distribution vector) [27], cumomer [28] and bondomer [29].

The present work describes the implementation and validation of OpenFLUX. Specifically, we explain the tasks performed by OpenFLUX, provide an illustration of the model definition setup of a hypothetical metabolic model, and also describe the structure and contents of the resulting metabolic models. The software is then validated by reproducing published ^13^C MFA results.

## Methods

### Implementation of OpenFLUX

**Figure 1:**
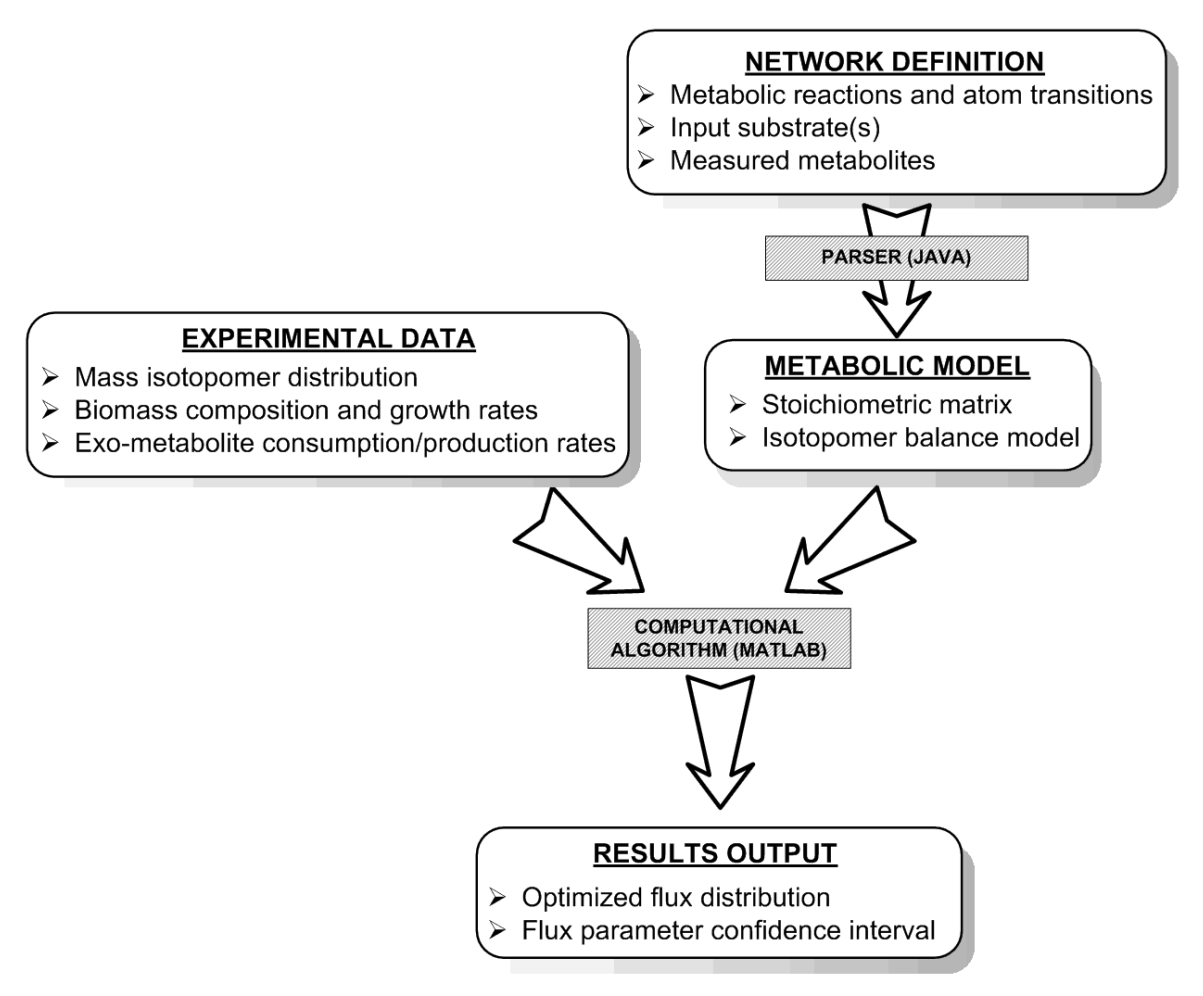
**Workflow of OpenFLUX**. The software consists of 2 components (shaded box) – a JAVA-based metabolic model parser and a set of MATLAB-based algorithms for parameter estimation and sensitivity analysis. The user defines the reaction network in the model definition file, which contains the metabolic model and the experimental data input. The parser reads the metabolic model, and subsequently generates a metabolite balance model (stoichiometric matrix), and an isotopomer balance model using the EMU framework. The MATLAB-based computational algorithms use these models, in conjunction with the experimental data, to perform least-square parameter estimation and parameter sensitivity analysis. The optimized flux distributions and confidence intervals are obtained as results output.

OpenFLUX consists of two parts for (i) automated model set-up or modification from user entered reaction data and (ii) the application to flux analysis by calculating fluxes from experimental data as well as statistical evaluation (Figure 1). The first part consists of a parser that automatically generates the metabolite and isotopomer balance models from a text-based model definition. The second part is a structured workflow that implements a series of numerical optimization algorithms for flux parameter estimation and sensitivity analysis.

#### Automated generation of metabolic models

OpenFLUX parses the metabolic model from a structured tab-delimited text file, created using a spreadsheet program, into MATLAB readable metabolite and isotopomer balance models. The former is the stoichiometric matrix (*S*) in the mass balance

(1)

while the latter is a non-linear function (*F*) that maps input substrate(s) mass isotopomer distribution(s) (MID) () to the simulated MIDs () using the flux distribution () as a parameter

(2)

The generation of the isotopomer balance model closely follows the recently developed EMU framework [25], whereby the model generation algorithm progressively constructs EMU balances by identifying all reactant EMUs that contribute to a given product EMU. The algorithm progresses until each of the simulated EMU products can be traced to the input substrate EMUs via a series of balancing equations. These equations are subsequently organized into matrix equations (see [25] for details).

#### Assignment of free fluxes

The solution to (1) can be expressed as a linear combination of free fluxes () [28]

(3)

where *NS *is the null space of S and the dimension of  equals the nullity of *S*, i.e., degrees-of-freedom in the system. It is these free fluxes that the optimization program will manipulate in order to achieve the best fit to the measured data.

The choice of free fluxes in (3) is not unique. In OpenFLUX, the flux vector is first decomposed into four different reaction types: bi-directional forward and reverse reactions ( and ), and irreversible reactions that are either free fluxes () or dependent fluxes (), and rearranged to the form

(4)

Using this form, the reduced column echelon form of the null-space matrix (*NS*)

(5)

explicitly maps the free fluxes defined as

(6)

to the full flux vector, .

This transformation confers two main advantages. Primarily, the assignment of free fluxes is automated to include all reverse fluxes and a subset of irreversible fluxes (6). The reverse fluxes are determined from the spreadsheet, where the user has identified the bi-directional reactions in the network, and, for each bi-directional reaction pair, the forward and reverse counterpart. Additional information may be specified, such as known reaction rates derived from experimental data (e.g., biomass precursor drain or extracellular rates), or the user's preference for an irreversible free flux assignment. OpenFLUX will then prioritize the assignment of free fluxes, but ultimately, the assignment is determined by the stoichiometric matrix.

A secondary advantage is that  is calculated explicitly from  in a single matrix operation (5). This circumvents the use of the stoichiometric matrix as an implicit constraint during optimization [17]. The formulation of the equation system used for calculation of absolute fluxes is greatly simplified compared to the convention proposed by Wiechert and de Graaf (1997) [28]. Furthermore, analytical derivation of the gradient matrix is straightforward (i.e., ).

We note that the flux coordinates for bi-directional fluxes, *v*^← ^and *v*^→^, while representing a natural choice, differ from the conventional flux coordinates, net flux (*v*^*net*^) and exchange flux (*v*^*xch*^), used in flux analysis [28]. Both flux coordinates can be compactified to the same effect whose main purpose is to improve the numerical performance. The rationale for the change in coordinates is detailed in the Appendix. It is straightforward to interchange between the coordinates using:

(7)

(8)

#### Flux calculation via numerical optimization

OpenFLUX uses FMINCON, a gradient-based minimization search function contained in MATLAB's Optimization Toolbox, to perform both flux parameter estimation and sensitivity analysis. FMINCON utilizes a quasi-Newton sequential quadratic programming (SQP) method for constrained, non-linear optimization. The algorithm is well-suited for metabolic flux analysis where physiologically meaningful boundaries exists for the free fluxes [22,30].

**Figure 2:**
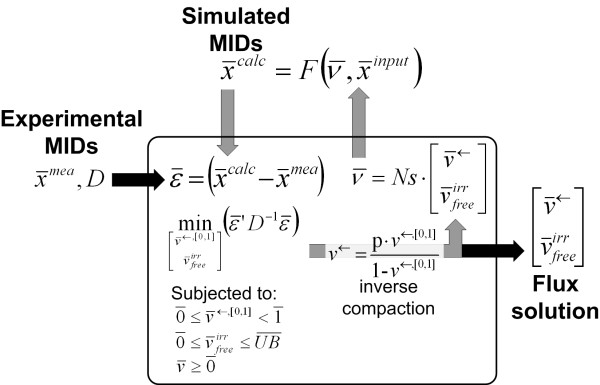
**Algorithm for weighted least-square parameter estimation**. OpenFLUX uses MATLAB's FMINCON to minimize the residual error () between the experimental MIDs () and the simulated MIDs (), by optimizing the free fluxes (), which are subjected to lower and upper boundary value constraints.  is weighted by the variance matrix (***D***). The inverse compactification function is used to transform reverse flux from a numerical parameter (*v*^←, [0,1]^) into a physical flux parameter (*v*^←^). The null-space matrix (*Ns*) is then used to calculate the flux vector () from the free fluxes (). Finally,  is calculated using  and known MIDs of the input substrates (). FMINCON terminates when a local minimum is found, revealing the optimum free fluxes.

For parameter estimation (Figure 2), the program searches for values of the free fluxes within the domains of the flux constraints in order to minimize the weighted sum of squared residual errors () between experimental data () and calculated values (), where the weight is the inverse of the variance-covariance matrix (**D**)

(9)

The program assumes measurements to be uncorrelated, i.e., **D **contains as a diagonal the individual measurement variances (). The constraints applied to the optimization are based on the definition that all fluxes must be positive, since reversible reactions are separated into the forward and reverse components

(10)

The optimization is also bounded to improve numerical stability. The irreversible fluxes  are non-negative and upward bounded by the net flux in the system. The upper bound (UB) is by-default set to 20 times the maximum substrate uptake rate, but can be varied

(11)

The reverse free fluxes  are non-negative, but have no natural upper limit. Hence, a compactification operation is used to transform from a physical [0, infinity] scale to a numerical [0, 1] scale [28].

(12)

A scaling factor (P) is used, whose value is typically of the same order-of-magnitude as the largest input substrate flux [31]. Alternatively, P can be adjusted during optimization to obtain a better conditioned Jacobian matrix [24].

#### Statistical evaluation of flux data

Flux statistics, e.g. confidence intervals of single flux parameters, are important to evaluate flux data and determine if observed differences between mutants or culture conditions reflect physiological differences. Confidence intervals for flux parameters (*Para*_*i*_) are determined using the non-linear approach developed by Antoniewicz et al. (2006) [30]. This approach provides a more accurate estimate of flux uncertainty than local estimates of the standard deviations. Briefly, the approach employs that the minimized variance-weighted sum of squared residual is approximately χ^2 ^distributed and therefore the difference (Δε;) between the objective function evaluated at the optimal solution and the objective function when one flux is fixed follows a χ^2^-distribution with one degree of freedom. Accordingly, an approximate (1-α) confidence interval for *Para*_*i *_can be defined by the two solutions for *para*_io _to

(13)

FMINCON is used to search for the solution pair, and each search is initialized from the optimum solution found during parameter estimation. The constraints applied in parameter estimation, (10)-(12), are also applied here ensuring that the boundaries to confidence interval represent feasible solutions. Analysis can be performed on different forms of *Para*_i_, such as free or dependent flux, flux ratio, or reversibility ratio. Alternatively, flux standard deviations can be estimated using the Monte Carlo approach included in OpenFLUX [32].

#### Flexible use for different types of experimental labelling data

OpenFLUX automatically indicates to the user the input substrate EMUs that must be specified prior to label simulation. Specifying these EMUs is done either by directly defining the vector elements of the input substrate EMUs, or indirectly using one of OpenFLUX's built-in functions. These functions calculate the corresponding vector elements using either the atom activity vector (AAV) or the isotopomer distribution vector (IDV) of the input substrates, both of which are well known notations in flux modelling [26,27].

The simulated output vector () can be modified by the user to suit the experimental data type. The mathematical manipulations that can be performed on  include (i) integrating mass interference from non-backbone stable isotopes using Cauchy-product [33], (ii) normalizing and truncating EMU variables to match the length of a shorter MID vector (typically 2 to 3 elements per metabolite), (iii) converting EMU variables into summed fractional labelling (SFL), and (iv) include or exclude specific element in an EMU variable (e.g. to represent positional enrichment data). These modifications are not automated by the software because they can be very diverse.

An EMU variable is essentially a MID vector. One can immediately simulate mass spectroscopy data using EMU variables. It is, however, possible to generate NMR fine spectra from EMU variables (detailed in [25]). OpenFLUX currently does not support automatic generation of NMR fine spectra, thus the user needs to list all essential EMU variables required for the transformation process during model set up and enter the transformations manually.

#### Design of experiments

Not all intracellular fluxes can be resolved in a single labelling experiment, but careful experimental planning can ensure that key unknown fluxes are determinable [34,35]. Determining the optimal labelled substrates to use and optimal metabolites to measure is a non-trivial task, often requiring sophisticated design strategies [31,36-38]. A common theme emerging from these design approaches is that one must be able to visualize the relationship between flux distribution, input substrates used and labelling response measured. OpenFLUX partially supports experimental design by allowing user to perform forward label simulation, that is to predict the labelling state of specified EMUs based on fluxes and input labels. Using Monte-Carlo simulation to explore fluxes in the expected range, it is possible to establish – for a given input label – what EMUs are most responsive to changes in flux, and hence what EMUs should preferably be measured.

#### Excluded metabolites and reactions

The spreadsheet based model specification allows the user to specify input substrate(s), simulated EMU variables as well as metabolites excluded from the stoichiometric matrix.

Five reaction types are supported. In addition to irreversible ("F") and reversible forward ("FR") and reversible reverse ("R") fluxes, it is possible to specify reactions used only for metabolite balancing ("B") and reactions used only for isotopomer balances ("S"). The atom transition equation for "B" type reaction is not required so that only relevant information has to be generated prior to the calculation. Type "S" reaction is a convenient approach to map a product's MID to their respective precursor(s) without incurring additional degrees-of-freedom in the metabolite balance model. Metabolites unrelated to the isotopomer balances, such as ATP and NADH, are marked with "X" in the atom transition equation. This allows user to set up a metabolic model that includes co-factor balances.

### Test case flux analysis TCA cycle

**Figure 3:**
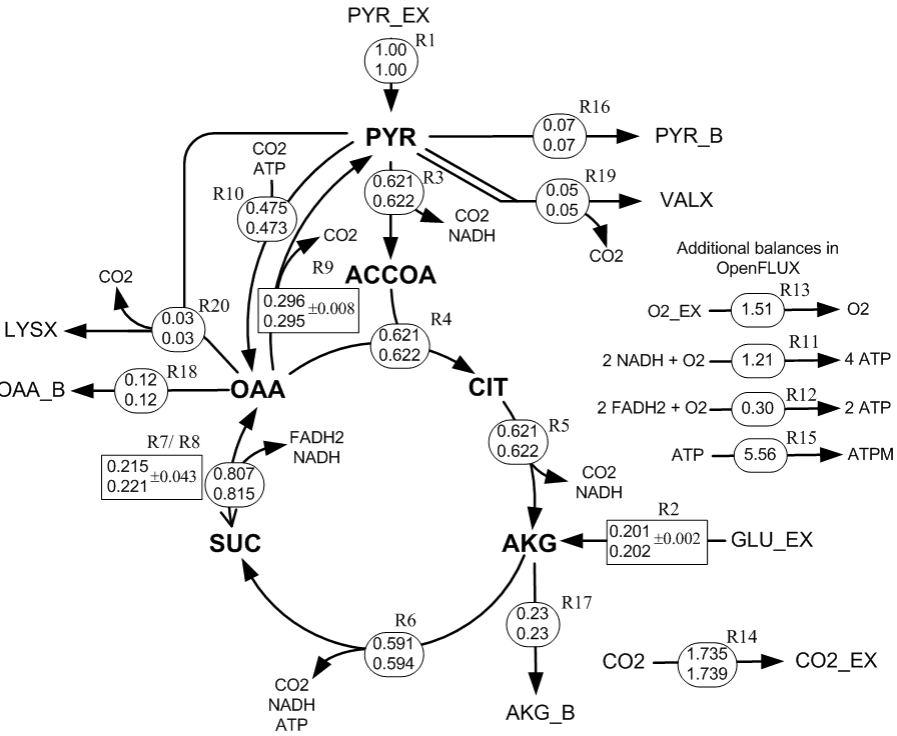
**Flux distributions for a simplified TCA cycle model as described in the text and Appendix**. Fluxes were calculated using OpenFLUX (top values) and the cumomer package 13C-FLUX [22] (bottom values). The absolute forward fluxes are displayed in the circles. The free fluxes (R2, R8, R9) and their 95% CI (generated by OpenFLUX) are displayed in the rectangular boxes. Full arrowhead: forward flux. Line arrowhead: reverse flux. All reactions are unidirectional except for the SUC↔OAA reaction. Suffix: _B, biomass drain; _EX, exo-metabolites. Metabolites: PYR, pyruvate; ACCOA, acetyl-CoA; CIT, citrate/isocitrate; AKG, α-ketoglutarate; SUC, succinate; OAA, malate/oxaloacetate; GLU, glutamate. Reactions: R1, pyruvate uptake; R2, glutamate take; R3, pyruvate dehydrogenase; R4, citrate synthase; R5, iso-citrate dehydrogase; R6, α-ketoglutarate dehydrogenase & succinyl-CoA hydrolase; R7/R8; fumarate hydratase & malate dehydrogenase; R9, malic enzyme; R10, pyruvate carboxylase; R11/R12, oxidative phosphorylation; R13, oxygen uptake; R14, CO2 evolution; R15, ATP maintenance; R16, pyruvate biomass drain; R17, α-ketoglutarate biomass drain; R18, oxaloacetate biomass drain.

**Figure 4:**
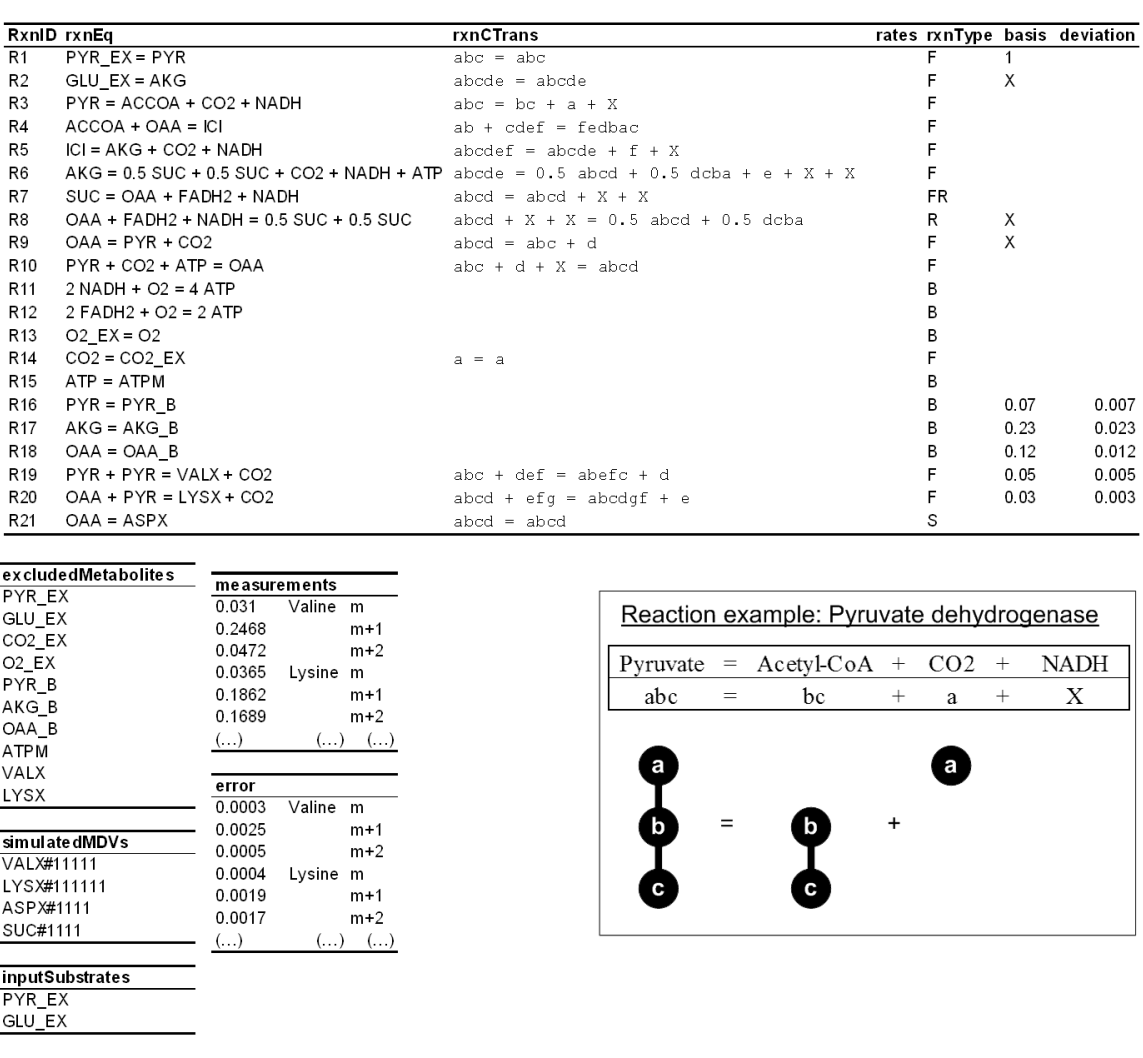
**Overview of the network model definition text file**. The model definition file consists of one table and five lists. The metabolic reaction network is defined in the table, and is organized under the following headings: reaction ID (*rxnID*), reaction equation (*rxnEQ*), reaction atom transition (*rxnCTrans*), reaction rate (rates), reaction type (*rxnType*), free flux allocation (*basis*) and flux value standard error (*deviation*). If a given reaction rate is known (e.g., biomass drain rates), then the flux value and the corresponding measurement error can be included in the *basis *and *deviation *columns respectively. The parser requires the user to separately list down metabolites that are excluded from the stoichiometric model (*excludedMetabolites*), EMUs that are to be calculated by the isotopomer model (*simulatedMDVs*), and input substrates that contribute to the isotopomer balance (*inputSubstrates*). The experimental MIDs (*measurements*) and the associated errors (*error*) are listed in the same order as the EMUs in the *simulatedMDVs *list. The figure insert (bottom right) is an example of how pyruvate dehyrogenase reaction is described in the *rxnEQ *and *rxnCTrans *columns. CO_2 _is produced by cleaving the carboxylic end (C1) of pyruvate, leaving the acetyl moiety (C2–C3). Alphabet letters in the atom transition equation is used to represent the transfer of first carbon atom of pyruvate to CO_2_, and the second and third atoms of pyruvate to the first and second atoms of acetyl moiety. An exception is the letter "x" or "X", which is used to exclude metabolite from the isotopomer balance, such as NADH.

OpenFLUX' semantics and algorithms were tested using a small network. The metabolic network is a representation of a condensed TCA cycle (Figure 3, 4), which also includes gluconeogenic (R9) and anaplerotic (R10) fluxes. The network has 2 input substrates (pyruvate (R1), glutamate (R2)), 3 biomass precursor drain fluxes (oxaloacetate (R18), α-ketoglutarate (R17), pyruvate (R16)), and 1 reversible reaction (R7 – forward, R8 – reverse) between succinate and oxaloacetate. Oxidative phosphorylation reactions are also included (R11, R12). It is assumed that the CO_2 _efflux (R14) is unidirectional [see Additional file 1]. The synthetic experimental data assumed available consists of the production rates of valine and lysine (R19 and R20), the precursor withdrawal rates from pyruvate, α-ketoglutarate and oxaloacetate towards biomass formation, and values on the relative fraction of the three mass isotopomers M_0_, M_1 _and M_2 _for valine, lysine, aspartate and succinate. The labelling data were corrected for mass interference from non-carbon backbone atoms. Fluxes were normalized to the pyruvate input flux of 1 mmol g^-1 ^h^-1^. A detailed instruction of how to formulate, set up and implement the model using OpenFLUX is provided in the Appendix.

### Metabolic model for lysine producing *Corynebacterium glutamicum*

We reconstructed the metabolic network of lysine producing *C. glutamicum *based on published information [39] and additional modelling details kindly provided by the authors [see Additional file 2]. The input substrate used was [1-^13^C]-glucose (with 99% enrichment purity), and all fluxes were normalized with respect to the glucose uptake rate (i.e., fluxes are expressed in percentage of glucose uptake rate). As the published MIDs are uncorrected, all the simulated EMU variables were modified for mass interference from non-carbon backbone isotopes using the molecular formula of the amino acids fragments (i.e., parent ion cluster). The first *n*+1 signal elements were normalized (*n *indicates number of backbone carbon), and then truncated to the correct vector length (equivalent to the measured MIDs) before performing weighted least-square analysis. The inferred metabolic model consisted of a total of 71 reactions and 42 balanceable metabolites. The metabolite model yielded a total of 26 degrees-of-freedom and 18 fluxes were determined experimentally: anabolic precursor yields (11), biomass yield (1), secreted product yields (5), and glucose uptake rate (1). To reduce the number of unknown parameters, these 18 fluxes were chosen as free fluxes, and the associated flux values were used deterministically as no redundant data exist in the measurement set. Note that if one suspects gross measurement errors in the flux measurement set, then these fluxes should be set free and the flux values subjected to the least-square analysis together with the MIDs. Five (5) of the remaining 8 free fluxes are associated with the reversibility of non-oxidative pentose-phosphate pathway enzymes (3), glucose-6P isomerase (1) and intercellular CO_2 _exchange (1). The other 3 free fluxes were assigned (by the software) to the irreversible fluxes of glucose-6P dehydrogenase, pyruvate carboxylase, and glycine synthesis via the serine route.

The MID of 9 amino acids and trehalose were reported. Three "S" type reactions were included in the metabolite network to directly map label distribution of alanine, aspartate and glutamate to pyruvate, oxaloacetate and α-ketoglutarate, respectively. This was not necessary for all other amino acids and trehalose because these metabolites were already described in the isotopomer balances.

### Computational requirements

All computational work was performed on a Pentium D 3.00 GHz computer. OpenFLUX was implemented in MATLAB 7.3.0.267 (R2006b) (The MathWorks, Natick, MA, USA). Numerical optimization was carried out using FMINCON function from MATLAB's Optimization Toolbox. Since we used numerical gradient, FMINCON automatically used the active-set algorithm (also known as "medium scale"). Termination tolerance on the function value was set to 1 × 10^-4^, and the optimizations were terminated when the magnitude of directional derivative in search direction is less than 2 × 10^-4^. 13C-FLUX was implemented in Ubuntu Linux 7.1 operating system.

## Results and discussion

### Test case flux analysis TCA cycle

**Figure 5:**
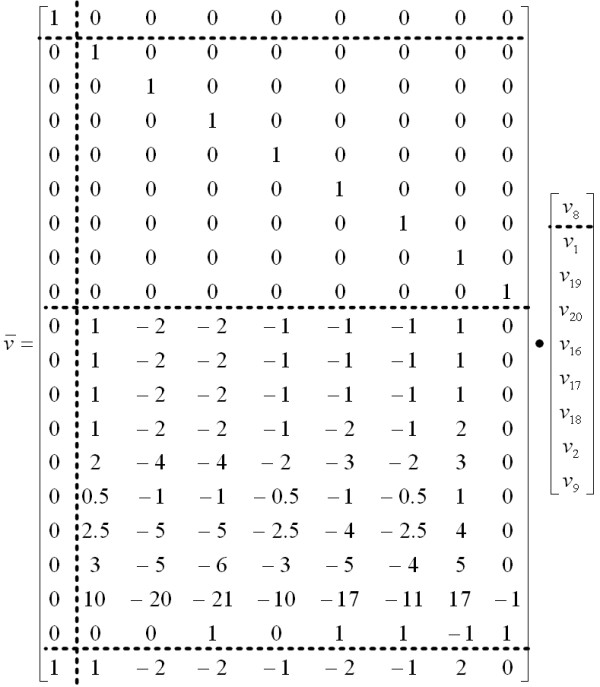
**The null-space matrix generated from the stoichiometric model**. The structure of this *NS *reflects the generic *NS *matrix form shown in Eq. 3. The Identity matrix indicate the 1-to-1 mapping of the 9 free flux parameters – ν_8_, v_2_, v_9 _are the unknown parameters, while ν_1_, ν_19_, ν_20_, ν_16_, ν_17 _and ν_18 _are the known parameters.

The specified network (Figure 3) has 9 degrees-of-freedom. Of the required 9 free fluxes, 7 were assigned by default from the definition: R1 (specified input flux), R8 (reverse flux), R16, R17 and R18 (specified biomass precursor drain fluxes), and R19 and R20 (specified amino acid production flux). R2 was specified as a preferred free parameter in the model definition, and – since this was an acceptable choice – was assigned by the parser. OpenFLUX automatically assigned the remaining free parameter to R9. Note that the correct *NS *structure (Eq. 3) is automatically produced (Figure 5).

The parser-generated isotopomer balance model consists of 50 EMU variables organized into 7 matrix equations, of which 14 are known input substrate EMUs. With the EMU approach, the total number of unknown scalar MID variables (116 variables) involved in the optimization is significantly less than a complete isotopomer model (262 variables).

The model created by OpenFLUX was validated for the TCA cycle test case by comparing the labelling data simulated for a given set of fluxes with cumomer based simulation (i.e., 13C-FLUX) [22]. Forward simulation based on hypothetical fluxes yielded identical labelling data results for all cases (data not shown). This means that the semantics used to specify the reaction network and the underlying isotopomer balance model constructed by OpenFLUX via the EMU decomposition algorithm is consistent.

The labelling patterns obtained for the test case were next corrupted with 1% Gaussian noise, and subjected to least-square analysis using 13C-FLUX [22] and OpenFLUX. Randomized initial solutions were used to start each optimization. All approaches yielded identical optimum solutions, consistent with the hypothetical values (Figure 3). This is an essential performance benchmark, since new semantics were introduced to define reversible and scrambling reactions.

The introduction of "X" type metabolites and "S" type reactions in OpenFLUX' flagging system for metabolites and reactions has several advantages. In this example, OpenFLUX automatically calculated the cofactor balances and the required oxygen uptake rate based on a given P/O ratio of 3 (Figure 5). OpenFLUX could also use the labelling input from aspartate, without introducing this amino acid as a balanceable metabolite. With 13C-FLUX, we had to artificially create a new reaction withdrawing a fraction of oxaloacetate to constitute the flux of aspartate synthesis and allow the consideration of its labelling pattern for flux estimation.

### Real case flux analysis – lysine producing *C. glutamicum*

The performance of OpenFLUX was tested for a real case flux scenario, comparing a low and a high lysine producing mutant of the soil bacterium *C. glutamicum *[39]. For these mutants, fluxes were previously quantified combining mass balancing with tracer experiments on [1-^13^C]-glucose, GC-MS ^13^C enrichment analysis of proteinogenic amino acids [10,40], and isotopomer mapping matrices based simulation.

The isotopomer model for *C. glutamicum *is comprised of 108 unknown EMU variables, with 15 additional known [1-^13^C]-Glucose and CO_2 _EMUs. A total of 20 balancing matrix equation sets were used to calculate the unknown EMUs, 9 of which are single equation balances that are easily calculated. The 108 unknown EMU variables correspond to 360 scalar MID variables, which is a substantial reduction from the 8380 unknown scalar variables expected in the full isotopomer model.

A total of 50 cycles of optimization were performed for both wild type and engineered strain datasets. The total computation time for each cycle was about 16 seconds. The calculated MIDs shown in Table 1 were derived from one of the optimization cycles that showed the smallest weighted sum of square residuals. All of the MIDs calculated by OpenFLUX were consistent with the published data (Table 1). The optimized free fluxes were also reproduced (Table 2). Comparing the 90% confidence intervals reported by Becker et al. [39] with the gradient-based search intervals generated by OpenFLUX, we could infer that there is no significant difference between the estimated free fluxes. In addition to the gradient-based search, we used the Monte-Carlo approach to generate the 90% CI [32,41] (Table 2). Briefly, the analysis was carried out by corrupting both the MIDs measurements with the prescribed relative error and the reported experimental yields for biomass and by-products with the corresponding deviations. The new data set was then used for parameter estimation. Optimization was performed iteratively until a sample size of 150 independent flux distributions was obtained. The 90% CI for a single parameter was then calculated using the free parameter's mean and variance estimated from the samples [42]. The 90% CIs generated through Monte-Carlo simulation were consistent with the original work, as well as with the intervals generated by gradient-based search. Notably, the confidence interval generated by Monte Carlo approach is generally wider than the gradient-based search because the variations in the experimental yields were included in the analysis. The wider 90% CI managed to capture all of the published optimum free fluxes. Overall, this validates OpenFLUX's accuracy with respect to the isotopomer balance model and the numerical approach.

**Table 1:** Experimental and calculated MIDs of the wild-type and engineered *C. glutamicum*.

		Wild-type			Mutant		
Metabolite fragment		Published data		Present work	Published data		Present work
		Exp	Calc	Calc	Exp	Calc	Calc
ALA 260	M_0_	0.508	0.509	0.509	0.523	0.525	0.525
	M_1_	0.353	0.354	0.354	0.341	0.342	0.342
	M_2_	0.106	0.106	0.106	0.103	0.104	0.104
VAL 288	M_0_	0.345	0.348	0.348	0.364	0.366	0.366
	M_1_	0.398	0.398	0.398	0.392	0.392	0.392
	M_2_	0.184	0.184	0.184	0.175	0.175	0.175
THR 404	M_0_	0.333	0.334	0.334	0.344	0.344	0.344
	M_1_	0.376	0.376	0.376	0.373	0.371	0.371
	M_2_	0.196	0.196	0.196	0.191	0.192	0.192
ASP 418	M_0_	0.334	0.333	0.333	0.345	0.343	0.343
	M_1_	0.373	0.375	0.375	0.370	0.370	0.371
	M_2_	0.195	0.196	0.196	0.192	0.193	0.192
GLU 432	M_0_	0.247	0.25	0.249	0.257	0.264	0.264
	M_1_	0.365	0.366	0.366	0.365	0.365	0.365
	M_2_	0.241	0.239	0.240	0.236	0.232	0.232
SER 390	M_0_	0.450	0.449	0.448	0.462	0.463	0.463
	M_1_	0.358	0.358	0.358	0.349	0.349	0.349
	M_2_	0.143	0.143	0.144	0.140	0.140	0.140
PHE 336	M_0_	0.271	0.274	0.274	0.287	0.289	0.289
	M_1_	0.382	0.381	0.381	0.380	0.381	0.381
	M_2_	0.228	0.228	0.228	0.220	0.220	0.220
GLY 246	M_0_	0.741	0.742	0.742	0.741	0.743	0.743
	M_1_	0.184	0.185	0.185	0.183	0.184	0.184
TYR 466	M_0_	0.234	0.236	0.236	0.246	0.249	0.249
	M_1_	0.353	0.356	0.356	0.351	0.358	0.357
	M_2_	0.242	0.245	0.245	0.234	0.238	0.238
TRE 361	M_0_	0.061	0.062	0.062	0.088	0.088	0.088
	M_1_	0.604	0.607	0.606	0.573	0.577	0.574
	M_2_	0.207	0.207	0.207	0.213	0.213	0.213

Sum weighted residues*			761	684		1735	1461

* For comparative reasons, residuals resulting from minuscule technical replication errors used in original work, were accepted here.

Table contains experimental and calculated MIDs published in Becker et al. [39]. The MIDs generated using OpenFLUX are consistent with the original data. The MIDs are uncorrected and have been normalized to the sum of the first n+1 peaks (n = number of backbone carbon).

**Table 2:** Estimated optimum free fluxes (mmol/100 mmol Glucose) and the associated 90% confidence interval.

		Wild-type		Mutant	
Flux parameter		Optimum	90% CI	Optimum	90% CI

Glucose-6P dehydrogenase	A	46.8	[46.3, 47.3]	56.2	[55.9, 56.5]
	B	46.7	[46.3, 47.1]	56.3	[56.1, 56.5]
	C		[46.2, 47.3]		[56.0, 56.6]
Pyruvate carboxylase	A	71.5	[67.6, 75.7]	62.6	[60.0, 64.8]
	B	74.9	[70.9, 79.3]	63.6	[61.8, 65.6]
	C		[70.4, 79.5]		[61.2, 65.7]
Glucose-6P isomerase (rev)	A	1.3	[1.2, 1.3]	2.4	[2.3, 2.4]
	B	1.27	[1.26, 1.30]	2.37	[2.33, 2.39]
	C		[1.24, 1.31]		[2.32, 2.41]
Transketolase 1 (rev)	A	0	[2.1, 2.5]	0	[0, 0]
	B	2.21	[2.03, 2.35]	0	[0, 0]
	C		[1.93, 2.49]		[0, 0]
Transaldolase (rev)	A	2.2	[2, 2.3]	4.5	[4.3, 4.6]
	B	2.16	[2.06, 2.24]	4.50	[4.40, 4.62]
	C		[2.00, 2.33]		[4.31, 4.69]
Transketolase 2 (rev)	A	1	[0.7, 1.3]	1.6	[1.4, 1.7]
	B	1.02	[0.84, 1.21]	1.50	[1.44, 1.57]
	C		[0.73, 1.41]		[1.41, 1.61]

A. Original work [39]; B. Gradient-based search; C. Monte Carlo. B's and C's optimum values are derived from the best optimum solution found by OpenFLUX. The unit for fluxes and confidence intervals is mmol/100 mmol glucose.

## Conclusion

OpenFLUX is a generic and efficient novel software tool for ^13^C MFA. We have shown that the front-end model setup is relatively simple and intuitive, and the back-end optimization is stream-lined and is significantly faster than 13C-FLUX. Overall, this means that studying large-scale metabolic models becomes more tractable, especially for problems that have large number of unknown free fluxes and/or demand more stringent statistical evaluation. The underlying metabolic models are transparent to the user, and could be adapted for other purposes. In addition, the definition of the simulated output vector is flexible, thus various experimental data types can be applied. Finally, the inclusion of two different but complementary confidence interval determination algorithms (i.e., non-linear and Monte Carlo) enables a more robust evaluation of flux solutions.

OpenFLUX performed well on real metabolic problems. We have applied the *C. glutamicum *metabolic model as an example problem, and were able to reproduce the calculated MIDs, the optimized flux parameters and the corresponding confidence intervals. We also showed that OpenFLUX is computationally fast.

OpenFLUX is useful for exploring different network topology, flux distribution and modelling assumptions. The application grants the user the ability to control the underlying metabolic models and data inputs via a simple textual interface. Experimental design is critical to justify choice of substrates and analytical techniques. One can troubleshoot potential observability and sensitivity issues by simulating hypothetical MIDs data on a typical range of flux distributions, and subsequently explore the resolution of different flux parameters. OpenFLUX supports testing of various labelled substrates. For incomplete metabolic models, ^13^C MFA could be used to validate soft assumptions regarding P/O ratio and cofactor balances (ATP, NAD(P)H).

Overall, the purpose of OpenFLUX is to encourage the adoption of ^13^C tracer studies for flux analysis in newly arising systems approaches. Providing it *open source *aims on further development of the software as a general platform for ^13^C fluxomics. ^13^C MFA requires significant upfront investment to construct the isotopomer balance model and to establish the numerical optimization for flux analysis. Users who are already familiar with MFA will find that ^13^C MFA is readily implemented once the atom transitions are included into the metabolic model. Lastly, the text-based spreadsheet interface is an effective means of disseminating the metabolic model because the network topology and modelling assumptions are readily found in a single model definition file.

## Symbols and abbreviations

AAV: atom activity vector; EMU: elementary metabolite unit; GC: gas chromatography; IDV: isotopomer distribution vector; MFA: metabolic flux analysis; MID: mass isotopomer distribution; MS: mass spectrometry; NMR: nuclear magnetic resonance; TCA: tricarboxylic acid; : weighted sum of squared residual errors; *I*: Identity matrix; *NS*: null space matrix; P: compactification scaling factor; *S*: stoichiometric matrix; WSSR: weighted sum of squared residuals; : measurement variances; : flux vector; : free flux vector; *v*^*net*^: net flux; *v*^*xch*^: exchange flux; *v*^←^, : reverse flux (scalar, vector); *v*^→^, : forward flux (scalar, vector); *v*^→, *net*^: net forward flux; : irreversible free flux vector; : irreversible dependent flux vector; *UB*: flux upper boundary; : simulated MID; : input substrate MID; : measurement MID.

## Availability and requirements

OpenFLUX is optimized for MATLAB 7.3 (R2006b) (The MathWorks, Natick, MA, USA) and Java 6 (Sun Microsystems, Santa Clara, CA, USA) on a Microsoft Windows XP platform. It requires the MATLAB Optimization Toolbox. The software is also available for MacOS 10.4.11 (and later) and Ubuntu linux 7.1 operating systems. Microsoft Excel 2003 was used to generate the model input for the Java parser, alternative spreadsheet programs are also useable.

OpenFLUX is released as open source and is available upon request, both compiled and as source code. Supplementary information is available online .

## Competing interests

The authors declare that they have no competing interests.

## Authors' contributions

LEQ programmed the JAVA parser, implemented OpenFLUX in MATLAB and performed the EMU based simulations. He also performed 13C-FLUX simulations and contributed to writing the paper. CWI performed the simulations based on isotopomer mapping, provided data and modelling details on the real test case scenario, contributed expertise for development of OpenFLUX and contributed to writing of the paper. LKN provided his expertise in mathematical modelling of fluxes and contributed to writing the paper. JOK designed and supervised the study. He contributed expertise in ^13^C fluxomics and coordinated the writing of the paper. All authors have approved the final manuscript

## Appendix

### Handling bi-directional reaction using a new flux coordinate

**Figure 6:**
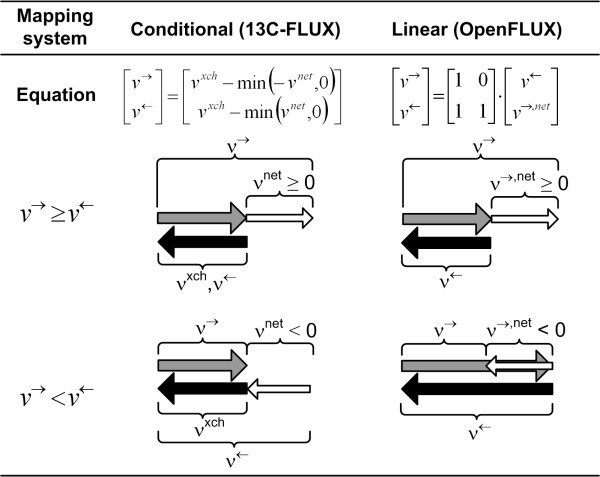
**Coordinate systems used to describe bi-directional reactions**. The schematics under the matrix equations shows how the two coordinate systems, [*v*^*xch*^, *v*^*net*^] and [*v*^←^, *v*^→, *net*^], are mapped to [*v*^←^, *v*^→ ^]. The former requires a conditional operator, which cannot be implemented using the null-space matrix. The prerequisite for the compactification transformation is that the black and grey arrows are overlapping.

The conventional coordinate system used to describe bi-directional reactions prevents the direct implementation of the null-space matrix [28]. In this system the net flux (*v*^*net*^) and exchange flux (*v*^*xch*^) are used as flux coordinates, where *v*^*xch *^is mapped to reverse flux (*v*^←^) if *v*^*net *^is positive, otherwise *v*^*xch *^is mapped to the forward flux (*v*^→^) (Figure 6).

The *v*^*net *^and *v*^*xch *^coordinate system was created for compactification purposes. As *v*^*xch *^can range from 0 to infinity, which represents either irreversible or fully reversible reactions, *v*^*xch *^is compactified using (12). This has been shown to reduce the response curvature of the ^13^C label with respect to the reaction's reversibility and improve linearization of the model [31]. However, such coordinate system invokes a complicated equation system to calculate  from [28].

Here, we introduce a straightforward linear coordinate system to describe bi-directional flux: *v*^→ ^is expressed as *v*^← ^plus a net forward flux (*v*^→^, ^*net*^), which can be positive or negative (Figure 6). This linear transformation is directly implemented within *NS *with a specific structure (5), which is readily created using Gauss-Jordan elimination. According to (5), we find that  and , i.e., *v*^→^, ^*net *^of a given reversible reaction is a linear combination of . This saves us from creating additional equation systems, as seen in the transformation of *v*^*xch *^and *v*^*net *^into *v*^→ ^and *v*^←^. *NS *with the required structure can be reproduced for all metabolic network configurations.

The new *v*^→^, ^*net *^and *v*^← ^coordinate system still allows the compactification of *v*^← ^to the same effect as compactification of *v*^*xch*^; since *v*^←^, like *v*^*xch*^, is mapped equally to the forward and reverse fluxes (Figure 6). However, there is one caveat with the coordinate system adopted by OpenFLUX. *v*^→ ^is forced to be a free flux after Gauss-Jordan elimination if all reactions in a branching pathway are reversible. To meet the conditions required for compactification, the null-vector associated with *v*^← ^is added to the null-vector associated with *v*^→^. As a result, *v*^→ ^becomes *v*^→^, ^*net*^, but *v*^→^, ^*net*^'s feasible range span both positive and negative domains. OpenFLUX has an algorithm to detect such occurrences and performs the appropriate modification to the free flux boundary value (i.e., -*UB *≤ *v*^→^, ^*net *^≤ *UB*).

### How to set up a model in OpenFLUX – test case central metabolism

#### Model setup in spread-sheet mode

In OpenFLUX, the metabolic model is organized into a table consisting of seven columns, three metabolite lists and two experimental data columns (Figure 4) [see Additional file 1]. The metabolic model configuration and experimental data input are gathered within a single spreadsheet interface, which contains all the information required to perform flux analysis. The parser only reads key active zones of the spreadsheet, thus other annotation information and user comments can be included in the spreadsheet. The first column contains the reaction ID. Arbitrary values can be assigned. It is important to note that the flux vector  in MATLAB is organized in the same order as presented in the table (i.e., the v(1) variable represents first reaction flux in the table). The second and third columns contain the reaction equations and corresponding atom transitions. Beforehand, it is important to distinguish reactions significant to the isotopomer balance from ones that are not. The definition of the latter reactions is flexible, and can accommodate floating-point coefficients, alpha-numeric metabolite names and an unlimited number of metabolite species.

#### Notation of metabolic reactions

The setup of the reactions relevant to isotopomer balance must obey the following rules: (1) reactant stoichiometry is always one; (2) same stoichiometric coefficients in both the reaction and atom transition equation; (3) only uni- or bi-molecular reactions. Within these rules, both normal and scrambling reactions can be adequately described. These rules imply that scrambling occurs on the product side of the reaction equation (i.e., R6 and R8). The limitation of reaction order to bi-molecular reaction is because the parser is not capable of generating a nested Cauchy-product for higher order condensation reactions. Nonetheless, higher order reactions can be decomposed into bi-molecular reactions. The Cauchy-product is the convolution of 2 sequences A and B, where the n^th ^element of the product is obtained from

(A1)

In the ^13^C tracer context, these sequences relate to the MID of 2 EMUs involved in a condensation reaction (see [25] for an example). A Cauchy-product function has been built into OpenFLUX.

The semantics for the atom transitions in the third column is based on a common convention [22,25], alphabetic notations are used to identify a series of carbon atoms in a metabolite, and are used to describe the transfer of the carbon atoms from the reactant to the product [26]. As long as the convention is consistently applied, different conventions could be used to describe the order of carbon atoms, typically seen in different automated carbon fate mapping algorithms [17]. A table of atom transitions in central carbon metabolism is provided [see Additional file 3]. The fourth column is used to display the flux distribution, and is only used for forward simulation of MIDs from the flux distribution (hypothetical or known).

#### Specification of reaction types of reaction reversibility

The fifth column contains the definition of each reaction type. There are 5 reaction types, namely "F", "FR", "R", "B" and "S". Reversible reactions are decoupled into the forward ("FR") and reverse ("R") flux. This scheme ensures that "R" type reactions (R8) are always assigned as free fluxes. "B", "S" and "F" are used to identify the relevance of a reaction to metabolite balance, isotopomer balance or both respectively. For "F" type reactions, metabolites that are to be excluded from the isotopomer balance are marked with "X" (case insensitive) in the atom transition equation. This is typically used for cofactors, such as NAD(P)H, FADH_2 _and ATP. Biomass drain fluxes (R16, R17, R18) and reactions that do not contribute to the tracer distribution (R11, R12, R13, R15) are classified as type "B" reactions. The atom transition equation for this reaction type is not required. Type "S" reaction is a convenient approach to map a product's MID to their respective precursor(s) without incurring additional degrees-of-freedom in the metabolite balance model, such as R21 for production of aspartate from oxaloacetate. This is especially useful when the labelling for a metabolite can be measured, but its production rate is unknown. Aspartate production rate is typically incorporated into the oxaloacetate biomass drain flux (R18).

#### Allocation of free fluxes

The sixth column contains the allocation of free fluxes for the metabolite balance model. It allows the user to specify an invariant value for a known free flux, or to allocate the preference for a reaction to be used as a free flux. For example, the drains of pyruvate, α-ketoglutarate and oxaloacetate to biomass were given the values of 0.07, 0.23 and 0.12 respectively. Additional drains for valine and lysine were added to enable subsequent model validation in 13C-FLUX. Also, the activity of pyruvate uptake flux is 1 because all fluxes are normalized to this reaction. "X" is assigned to R2 as a preferred free parameter as the reaction represents the glutamate uptake flux. By convention, "X" is assigned to any "R" type reaction.

#### Consideration of experimental noise for flux calculation

The seventh column carries the experimental standard deviations associated with each of the known fluxes. This allows a known flux value to be used either deterministically, where the flux value is fixed, or as an experimental measurement, where the flux is set free and the flux value is included in the least-square analysis. Using flux values deterministically may lead to gross-measurement error, but can reduce computation time.

#### Listing unbalanced metabolites, simulated EMU variables and input substrates

Metabolites that are considered external to the system must be identified in order to generate a balanceable stoichiometric matrix. The list comprises the reactants of the system inputs and products of the system outputs. The EMU variables to be simulated were chosen to be the full carbon backbone of valine, lysine, aspartate and succinate. They are written in the form "metabolite name#binary number". "1" and "0" are used to indicate whether the carbon atom at a given position is to be included or omitted respectively. For example, the simulated valine EMU variable is written as "VALX#11111". The experimental measurements and the associated errors are listed in the same order used to list the simulated EMU variables in the model definition file. The input substrates are pyruvate and glutamate, and are placed into another list. Generically, an input substrate is any exo-metabolite that enters the system boundary and can contribute to ^13^C isotopomer distribution in metabolites. The ^13^C enrichment of these metabolites must be known.

#### Error checking

Before the metabolite and isotopomer models are generated, OpenFLUX inspects the model definition file for potential inconsistencies. Mainly, OpenFLUX checks for consistency in metabolite naming, stoichiometry, carbon atom length for a given metabolite, and for presence of higher order reactions or unbalanced atom transition equation. The inconsistencies are reported to the user, and must be resolved before the metabolic models are successfully generated.

#### Parameter estimation and sensitivity analysis

Parameter estimation and sensitivity analysis is executed from MATLAB's command line. OpenFLUX is initialized by typing "start13OF". The user then chooses the various optimization tasks to be performed. After choosing the task, a series of command line prompts are given to the user to specify the parameters required by the optimization program. Once completed, the optimization begins.

## Supplementary Material

Additional file 1 **TCA cycle metabolic model**. Contains the hypothetical TCA cycle metabolic network, produced in the format that is readable by OpenFLUX. Information on how to set up the input substrates and the simulated output EMU vector are included. The EMU balance model used for flux calculation is also included. Click here for file

Additional file 2 ***C. glutamicum *metabolic model**. Contains the *C. glutamicum *metabolic network for both wild-type and mutant strains. Model and data are reproduced from Becker et al. [39]. File includes brief instruction on how to set up the simulated output EMU vector. Click here for file

Additional file 3 **Atom transition equations**. Contains typical reaction and atom transition equations encountered in the central metabolism. Pathways included are glycolysis (Embden-Meyerhof and Entner-Doudoroff pathways), TCA cycle (including glyoxylate shunt and anaplerotic reactions), pentose-phosphate pathway and amino acid biosynthesis from central metabolite precursors. Click here for file
